# Proposal and Definition of an Intelligent Clinical Decision Support System Applied to the Prediction of Dyspnea after 12 Months of an Acute Episode of COVID-19

**DOI:** 10.3390/biomedicines12040854

**Published:** 2024-04-12

**Authors:** Manuel Casal-Guisande, Alberto Comesaña-Campos, Marta Núñez-Fernández, María Torres-Durán, Alberto Fernández-Villar

**Affiliations:** 1 Fundación Pública Galega de Investigación Biomédica Galicia Sur, Hospital Álvaro Cunqueiro, 36312 Vigo, Spain; 2 NeumoVigo I+i Research Group, Galicia Sur Health Research Institute (IIS Galicia Sur), SERGAS-UVIGO, 36312 Vigo, Spain; marta.nunez.fernandez@sergas.es (M.N.-F.); jose.alberto.fernandez.villar@sergas.es (A.F.-V.); 3 Department of Design in Engineering, University of Vigo, 36208 Vigo, Spain; acomesana@uvigo.es; 4 Design, Expert Systems and Artificial Intelligent Solutions Group (DESAINS), Galicia Sur Health Research Institute (IIS Galicia Sur), SERGAS-UVIGO, 36312 Vigo, Spain; 5 Pulmonary Department, Hospital Álvaro Cunqueiro, 36312 Vigo, Spain; 6 Centro de Investigación Biomédica en Red, CIBERES ISCIII, 28029 Madrid, Spain

**Keywords:** COVID-19, long COVID, expert systems, fuzzy logic, automatic rule generation, intelligent system, clinical decision support system, artificial intelligence, decision-making, Wang-Mendel

## Abstract

Long COVID is a condition that affects a significant proportion of patients who have had COVID-19. It is characterised by the persistence of associated symptoms after the acute phase of the illness has subsided. Although several studies have investigated the risk factors associated with long COVID, identifying which patients will experience long-term symptoms remains a complex task. Among the various symptoms, dyspnea is one of the most prominent due to its close association with the respiratory nature of COVID-19 and its disabling consequences. This work proposes a new intelligent clinical decision support system to predict dyspnea 12 months after a severe episode of COVID-19 based on the SeguiCovid database from the Álvaro Cunqueiro Hospital in Vigo (Galicia, Spain). The database is initially processed using a CART-type decision tree to identify the variables with the highest predictive power. Based on these variables, a cascade of expert systems has been defined with Mamdani-type fuzzy-inference engines. The rules for each system were generated using the Wang-Mendel automatic rule generation algorithm. At the output of the cascade, a risk indicator is obtained, which allows for the categorisation of patients into two groups: those with dyspnea and those without dyspnea at 12 months. This simplifies follow-up and the performance of studies aimed at those patients at risk. The system has produced satisfactory results in initial tests, supported by an AUC of 0.75, demonstrating the potential and usefulness of this tool in clinical practice.

## 1. Introduction

Long COVID [1,2,3,4]—also known as the post-acute sequelae of SARS-CoV-2 infection—is a condition that has become increasingly relevant since the onset of the pandemic due to its impact on affected individuals. The condition is characterised by persistent symptoms even after the acute phase of the disease has subsided. The symptomatology is diverse; in fact, more than 200 symptoms have been identified [2] and may include cognitive dysfunction, fatigue, headache, anosmia or dyspnea, among others. The identification of those individuals susceptible to develop long COVID is of great interest; however, it is a complex task. Several studies in the current literature aim to identify risk factors that may predispose individuals to the development of persistent symptoms, including sex [5,6,7,8,9,10,11,12,13] and age [8,12,13,14,15]. Several comorbidities have also been identified that may increase the risk of developing long COVID, such as obesity and various pathologies (e.g., respiratory pathologies, diabetes, hypertension, etc.) [5,6,9,12,15,16,17]. Some authors have also explored the impact of vaccination [6,18,19,20], as well as socio-economic factors [8,21].

In the context of long COVID and its various manifestations, it is crucial to highlight dyspnea as one of the predominant complications [22,23,24], given its close association with the respiratory nature of COVID-19 as well as its significant impact on the daily lives of affected individuals. Despite numerous studies, the identification of patients likely to experience dyspnea for a prolonged period after initial recovery (6 to 12 months) remains a challenge. The pre-identification of these patients is of great interest to clinicians, especially pulmonologists, as it would allow for closer follow-ups and targeted studies.

In general, the use of tools and mechanisms to facilitate decision making is becoming increasingly common in healthcare. Typically, these tools are based on the use of techniques from the field of artificial intelligence integrated as clinical decision support systems [25,26,27,28,29,30,31,32,33,34,35,36,37,38,39,40,41,42,43,44]. Regarding the prediction of long COVID, there have been some singular proposals supported by the use of machine learning models [45]. In particular, the National COVID Cohort Collaborative (N3C), with information on more than 8 million patients obtained from their electronic health records, has allowed the development of models and proposals based on artificial intelligence [46]. Based on the N3C, the work of Plaff et al. [47] proposes the use of the XGBoost model to identify patients suffering from long COVID. They considered patients over 18 years old who had tested positive for COVID-19 and for whom at least 90 days had elapsed since the onset of the disease. They obtained results with area under the curve (AUC) values of 0.92, 0.90 and 0.85 for the whole population, hospitalised patients and non-hospitalised patients, respectively. The work of Blessy Antony et al. [48] addresses the use of two learning models (logistic regression and random forest) to predict which patients might develop long COVID. They started from the N3C and included data on acute phase symptoms, COVID-19 treatment interventions, medications prescribed during the acute phase of the illness, comorbidities and demographic information. The results obtained highlight AUC values of 0.76 and 0.75 for logistic regression and random forest, respectively. Similarly, the work of Reme et al. [49] explored the use of learning models (LASSO and random forest) to predict long COVID after three months of positive COVID-19 testing. This study used a Norwegian database containing information on more than 200,000 patients, including demographics, socioeconomic aspects, history of previous health care use, as well as the individual’s viral variant and vaccination status. The results were supported by AUC values close to 0.78 in both cases. These studies highlight the usefulness of artificial intelligence, especially machine learning, to help identify potential cases of long COVID. On the other hand, in their work, Gupta et al. [50] address the development of a model for the identification of patients who, after suffering COVID-19, may present cardiac sequelae. To do so, they start from a database with 180 patients, on which they deploy a stacking ensemble, supported by Deep Neural Networks, with accuracy values of 93.23%. In the work of Patel et al. [51], they use the random forest model, acting as a binary long COVID classifier, on a dataset of various blood proteins. Of the total 2925 proteins, they identified 119 as relevant to differentiating a patient with long COVID from one without the condition. In their model, they had AUC values of 1 in their reported results.

From a clinical perspective, and in line with the above, most current research on long COVID takes a general approach without focusing on specific symptoms or conditions, with the notable exception of the study by Gupta et al. [50], which focuses on post-COVID cardiac sequelae. Although these general approaches may be beneficial, it is important to note that long COVID is a condition that encompasses more than 200 symptoms that do not affect all patients in the same way, nor are they equally relevant to specialists in different medical areas. In addition, most approaches address short-term predictions, 3 or 6 months, which may be limited for specialists. On the other hand, as noted, all the detailed works, as well as those considered in the reviews on artificial intelligence models in the context of long COVID [45], employ statistical learning approaches. Most learning-based approaches (such as random forest, neural networks, etc.) can be understood as black box models, and even after applying techniques and strategies to improve interpretability (such as SHAP), it is difficult to understand the reasoning behind the predictions beyond identifying the variables with the highest predictive power in the available dataset. In general, no proposal based on symbolic approaches has been observed in the current literature featuring all the advantages and benefits that could be derived from a knowledge base. The incorporation of a knowledge base is a differential issue; this is because it not only facilitates the structuring and transmission of knowledge between experts, but it is also essential to promoting explainability, understood as the ability to trace and understand the rules or logical processes that underlie the predictions or decisions of a system. This ensures that healthcare professionals can understand exactly how a particular conclusion has been reached, which goes far beyond identifying the most important variables in a prediction, thus ensuring transparency in the associated decision-making processes.

In this article, we present the proposal of a novel intelligent clinical decision support system applied to the prediction of dyspnea 12 months after an acute episode of COVID-19.

The contributions of this paper are:

- To introduce at a conceptual level the architecture of a new intelligent clinical decision support system applied to the prediction of dyspnea after 12 months of an acute episode of COVID-19.
- Starting from the SeguiCovid database, select a reduced set of variables that can be used for the prediction of dyspnea after 12 months of a COVID-19 episode.
- Develop a new architecture, supported by a cascade of expert systems, whose knowledge bases are automatically generated using the Wang-Mendel automatic rule generation algorithm [52].
- Implement the intelligent clinical decision support system through a software artefact and demonstrate its utility through a case study.

The paper is divided into five sections. Section 2 presents the conceptual design of the intelligent clinical decision support system, explaining and detailing the different stages involved. This is followed by its implementation through a software artefact. Section 3 presents a practical application case, while Section 4 discusses the presented system. Finally, Section 5 presents the conclusions and future lines of work.

## 2. Materials and Methods

### 2.1. System Definition

#### 2.1.1. Database Usage

The SeguiCovid database is used to define the intelligent system [53,54,55]. This database was created between 2020 and 2022 in the Pneumology Department of the Álvaro Cunqueiro Hospital in Vigo and includes 194 patients. It is important to clarify that this is not the general population, but patients with severe COVID-19 who required hospitalisation due to the development of pneumonia.

All patients in the database were followed up to assess their outcome at 3 and 12 months after the COVID-19 episode.

The database is extensive and includes a large number of variables, but it is important to note that many of these may not be relevant for predicting dyspnea 12 months after the COVID-19 episode. For this reason, the pulmonologists on the team selected the variables they considered most important based on clinical criteria. After this selection, general and anthropometric data (sex, age and body mass index), toxic habits (smoking), previous comorbidities (lung diseases, heart diseases, diabetes, arterial hypertension), severity of the disease (need for admission to the Intensive Care Unit-ICU-or Intermediate Respiratory Care Unit-IRCU), the situation at three months (presence of dyspnea, X-ray results, and change in pulmonary diffusion capacity) and the situation at 12 months (presence of dyspnea) were considered. Table 1 shows a summary of the variables selected by the pulmonologists, indicating their type (numerical or categorical). The categorical variables in the database have been treated as numbers due to their binary nature, i.e., they can only take on two values: 0 or 1. In the comment column of Table 1, an explanation of the meaning for each value is given; for example, for the variable “Smoking”, a value of 1 indicates that the patient smokes, while a value of 0 indicates that the patient does not smoke. In the case of “Severity of pneumonia”, a value of 1 is related to the admission in ICU as opposed to a value of 0, which is related to IRCU admission. The rest of the categorical variables have been recoded in a similar way, as shown in Table 1.

#### 2.1.2. Conceptual Design

**Figure 1:**
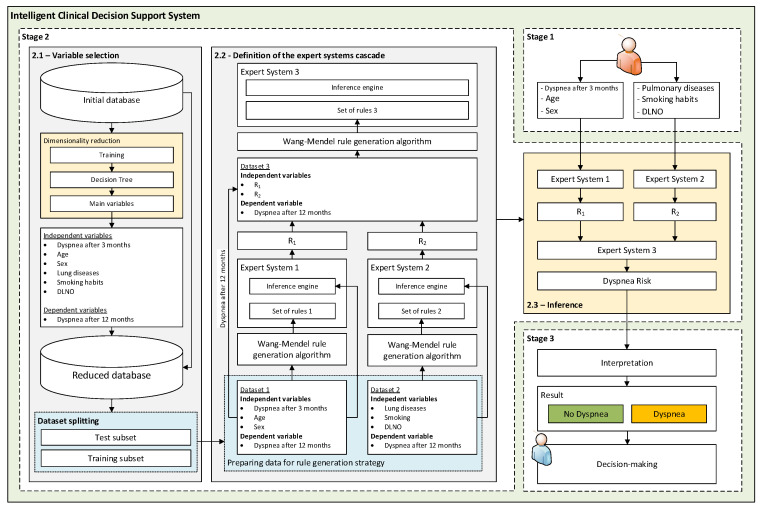
Flowchart of the Intelligent Clinical Decision Support System. In Stage 1, the collection of patient data is carried out. Stage 2 is subdivided into Stage 2.1, which focuses on the selection of the variables with the highest predictive power; Stage 2.2, which deals with the generation of the knowledge bases of the expert systems; and Stage 2.3, which deals with the symbolic inference process. Finally, Stage 3 deals with the generation of alerts and decision making.

A flow chart that illustrates the different stages of the intelligent system that is proposed in this paper is shown in Figure 1. A detailed description of each of these stages is given below.

##### Stage 1: Data Collection

The first stage involves the collection of the patient data previously introduced in Section 2.1.1. As will be explained later in Stage 2, not all the variables originally considered in Table 1 are used. Only sex, age, lung diseases, smoking and the presence of dyspnea in the third month after the COVID-19 episode and DLNO are collected.

##### Stage 2: Data Processing

After the collection and structuring of the patient data, the second stage is the processing of this data by the intelligent system. For this purpose, a set of cascaded expert systems is used. Below is a description of the different stages involved in the definition of the expert systems, from the selection of the input variables to the definition of the knowledge bases and the subsequent inference process.

- Stage 2.1—Selection of variables: Before building the knowledge bases of the expert systems, it is essential to determine the input variables for each of them. To identify the variables with a higher predictive power, it is proposed to use a CART-type classification tree [56,57]. The use of decision trees as a feature selection tool is a common practice and is supported by the scientific literature [58,59,60]. After this process, it is observed that, among the variables present in Table 1, those with the greatest predictive power are sex, age, lung diseases, smoking, presence of dyspnea in the third month and DLNO.
- Stage 2.2—Definition of the expert system cascade: Once the variables with the highest predictive power have been identified, their treatment by the system is addressed. For this purpose, a cascade of expert systems is used, all of them using fuzzy inference engines of the Mamdani type [61,62,63,64]. The cascade has two levels, as shown in Figure 1. In the first level of the cascade, Expert System 1 is fed with sex, age and dyspnea at month 3, while Expert System 2 is fed with lung diseases, smoking and DLNO, obtaining at its output the risks R_1_ and R_2_, respectively. Then, at the second level of the cascade, these risks are fed to Expert System 3, which obtains the *Dyspnea Risk* at 12 months. In this way, the use of the cascade reduces uncertainty and facilitates the creation of more accurate knowledge bases [38,65], with simpler rules due to the smaller number of antecedents. In general, one of the most complex tasks in the development of an expert system is the creation of the knowledge base. Although there have been various studies and proposals aimed at identifying the risk factors associated with the development of long COVID, there is currently no explicit knowledge or experience of how a patient might develop 12 months after the episode and whether he or she might present with dyspnea. Statistical approaches could be used to solve this problem, with the associated loss of explanatory power; however, in this work we opt to use the Wang-Mendel automatic rule generation algorithm [52]. In this way, starting from the dataset, it is possible to generate a knowledge base based on fuzzy rules for each of the expert systems.
- Stage 2.3—Inference: Once the expert systems are defined and given a new patient’s data, their treatment is approached and the *Dyspnea Risk* at 12 months is obtained.

##### Stage 3: Alert Generation and Decision-Making

Once the patient data has been processed, the *Dyspnea Risk* at 12 months is obtained at the output of the cascade. The final step is to interpret the risk indicator to obtain the final label associated with the patient (no dyspnea vs. dyspnea). Based on this information, the pulmonologist will be able to follow up with at-risk patients and conduct personalised studies.

### 2.2. System Implementation

This section deals with the implementation of the intelligent system presented in the previous section through a software artifact. For this purpose, MATLAB© software (version R2023b, Natick, MA, USA) is used together with Statistics and Machine Learning Toolbox [66] for training the CART model, the Fuzzy Logic Toolbox [67] for implementing the expert systems, and the App Designer Toolbox [68] for developing the graphical interface.

The equipment used to implement the system consists of an AMD Ryzen 9 7940HS processor with an NVIDIA GeForce RTX 4070 GPU graphics card and 32 GB of RAM.

**Figure 2:**
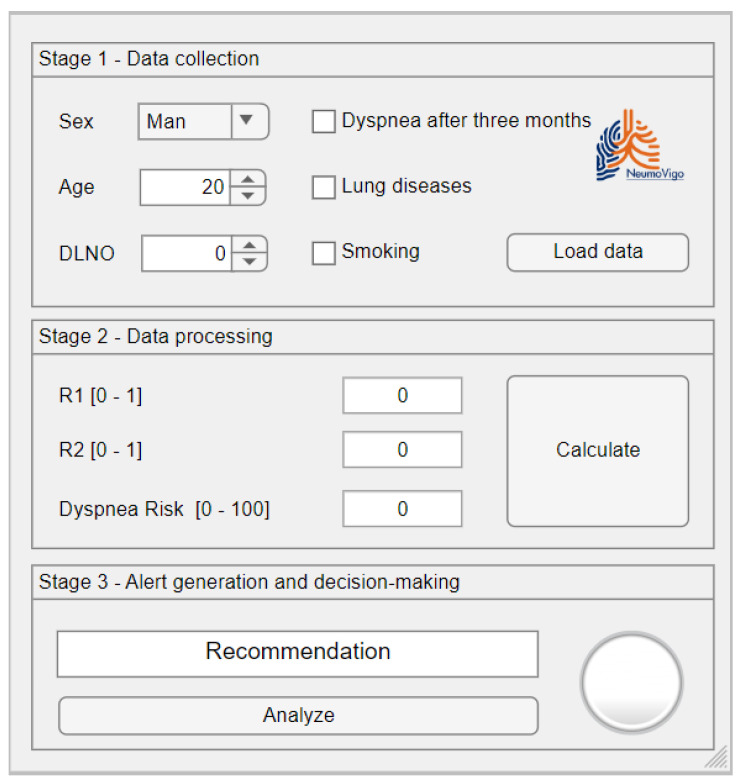
Screenshot of the main interface of the tool.

Figure 2 shows a screen shot of the graphical user interface of the tool that was developed. It shows three zones that correspond to the main stages of the intelligent system, as previously presented in Section 2.1.2.

#### 2.2.1. Data Collection

Patient data is entered using the form shown in Figure 2 in Panel (1), Data Collection. The medical team must check that the information entered is correct to avoid reducing the accuracy of the system and potentially increasing the associated uncertainty.

#### 2.2.2. Data Processing

Once the data has been entered, it is processed by the intelligent system. This process takes place in the (2) Data Processing panel, as shown in Figure 2.

##### Variable Selection

As mentioned above, prior to defining the expert systems, an analysis of the initial dataset was carried out to select the variables, also known as features in the context of machine learning, with the greatest predictive power.

In the area of feature selection, the objective is to identify an optimal subset of features that, during the model development phase, will help to simplify the model structure, enhance its interpretability, and reduce any redundancy between variables. There are various methods in this area, and they can be divided into filter methods, which dispense with the use of models and are based on statistical properties inherent to the data; wrapper methods, which incorporate predictive models to evaluate the relevance of different subsets of variables, selecting those that contribute to a significant improvement in the model’s performance (although this implies a higher computational cost); and embedded methods, which integrate the selection of variables as an essential component of the model development process, a characteristic that distinguishes them by their lower computational demands, given that the construction of the model is carried out only once.

Because of their computational efficiency and simplicity, in this paper we opt for the embedded methods through a CART-type decision tree.

It is important to note that no specific pre-processing of the data, such as rescaling, was carried out, as the model used, a CART-type decision tree, does not require it. Furthermore, in this initial phase, the complete data set was used.

For the construction of the tree, the Statistics and Machine Learning Toolbox was used, relying on the Gini measure as an impurity criterion and setting a maximum value of 9 splits to control the complexity of the tree. In a sense, this strategy aims to reduce the number of predictors, thereby simplifying the subsequent cascade structure and facilitating the creation of simpler knowledge bases.

After constructing the tree, the model drops some of the initial variables, retaining the presence of dyspnea at month 3, as well as age, sex, lung diseases, smoking and DLNO to address the prediction of dyspnea at 12 months after the COVID-19 episode.

When selecting these variables in the dataset, it is noted that some patients have missing fields in some of the variables, which is not a serious problem in the case of the decision tree. However, before constructing the cascade, these cases are discarded, leaving a total of 185 patients.

##### Definition of the Expert System Cascade

Once the variables with greater predictive power have been identified, the next step is to set up the cascade and determine the knowledge bases of each of the expert systems.

The strategy of using a cascade of expert systems has been introduced previously and is designed to reduce uncertainty and facilitate the creation of more accurate and simpler knowledge bases. Using a single expert system with six antecedents in its rules would be impractical, as it would result in an overly complex knowledge base, as well as no rules being fired in some cases, potentially.

As there are six antecedents, it was decided to define two expert systems at the first level of the cascade, with three antecedents per expert system. The outputs of these expert systems are in turn input to another expert system at the second level of the cascade, which determines the *Dyspnea Risk* at 12 months. A summary of the variables associated with each expert system is shown in Table 2.

Expert systems with fuzzy inference engines of the Mamdani type [61,62,63] are used. Their knowledge bases are determined using the Wang-Mendel automatic rule generation algorithm [52]. Regarding the datasets for each expert system, the independent variables are the input variables listed in Table 2, while the label, the dependent variable, is the presence of dyspnea 12 months after the COVID-19 event (expressed as 0 or 1).

Of the 185 available patient records, obtained after excluding patients with missing values, 30% were set aside for testing and evaluating the predictive ability of the system once it was built. The remaining 130 were used to build the knowledge bases of the expert systems.

**Figure 3:**
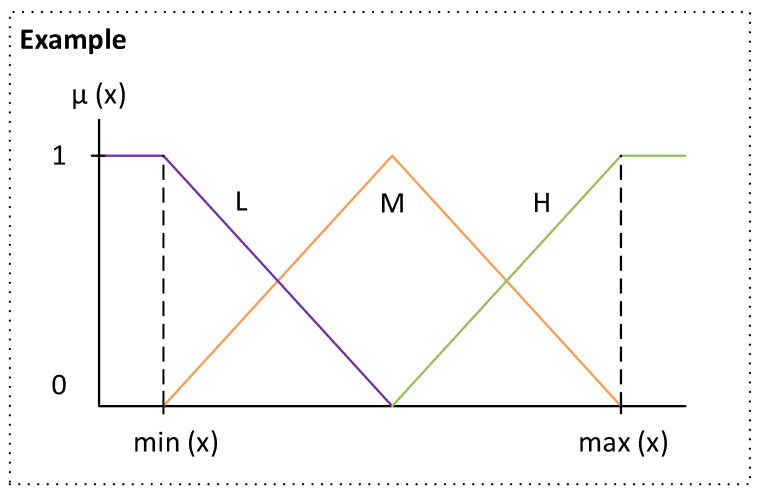
Example of division of the starting domain for a generic variable x with N = 1.

**Figure 4:**
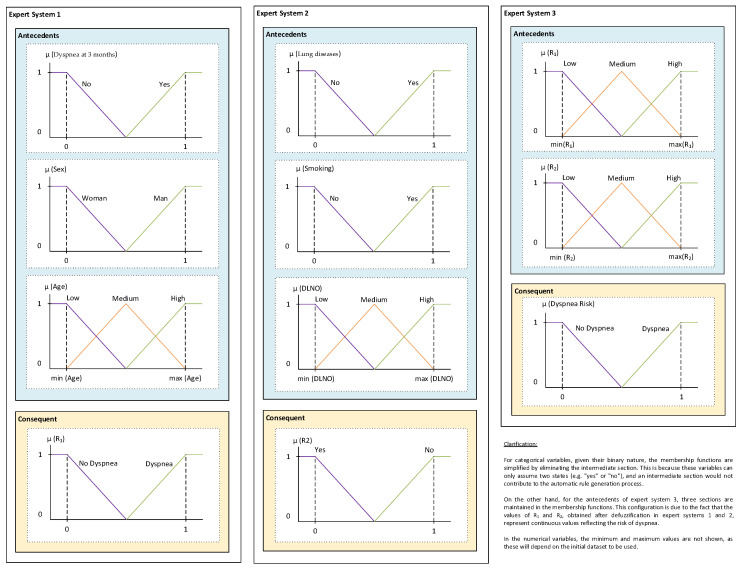
Division of the domain for the different variables.

The use of the Wang-Mendel automatic rule generation algorithm can be systematised in a series of steps [42,52], as described below, and adapted to each expert system:

- Step prior to applying the method: First, the shape of the membership functions is defined. Triangular membership functions are chosen for both antecedents and consequents, as in the original Wang-Mendel paper [52]. In addition, the value of a parameter N is defined, which is related to the number of sections that the membership function of each variable will have (the number of sections of the membership function is equal to 2N + 1, to ensure that there is a central section). In this case, a value of N = 1 is set for both the antecedents and the consequents, giving a total of 3 sections per variable. In the case of the categorical input variables (dyspnea at 3 months, sex, lung diseases and smoking) and the output variable (the label), although there are three sections, only the extreme ones are used (this is because there are two unique values: 0 and 1).
- Step 1—Division of the input and output spaces into fuzzy regions: After setting the algorithm configuration, the division of the initial spaces, both antecedent and consequent, is addressed using the parameters set in the previous stage. Figure 3 shows a generic case for N = 1 with three sections of the membership function (Low, Medium and High). In line with the proposal of the original Wang-Mendel paper, the overlapping of the triangles is considered so that, if the top vertex of the central triangle has a maximum membership degree, the vertices of the neighbouring triangles at the same point have minimum membership degrees. For each of the expert systems, Figure 4 shows the different associated membership functions. For categorical variables, given their binary nature, the membership functions are simplified by eliminating the intermediate section. This decision is based on the observation that, since these variables can only assume two states (e.g., “yes” or “no”), an intermediate section would not contribute to the automatic rule generation process, nor to the subsequent inference. On the other hand, for the antecedents of Expert System 3, which are obtained after the inference and defuzzification of Expert Systems 1 and 2, we have kept three sections in the membership functions. This is because the values of R_1_ and R_2_ are continuous values obtained after the defuzzification process in Expert System 1 and 2, respectively, reflecting the risk of dyspnea.

- Step 2—Generation of fuzzy rules: After the division of the starting spaces, we move on to the generation of fuzzy rules. For this purpose, the degree of membership associated with each of the sections of the membership functions for each line of the dataset is determined. After that, in each of the lines of the dataset, each variable is assigned to the section with the maximum degree of membership, determining a rule for each line. In this case, as there are 130 patients reserved for the construction of the model, 130 rules are initially obtained in each knowledge base. It is important to clarify that the knowledge base of Expert System 3 is constructed once those of Expert Systems 1 and 2 have been determined, as it is necessary to construct a derived dataset with the values of R_1_, R_2_ and the label from the initial data.
- Step 3—Assigning a degree to each rule to resolve conflicts: After generating rules in Step 2, it may happen that there are rules with the same antecedents but different consequents. To solve this problem, in the original proposal by Wang-Mendel [52], a coefficient is determined for each rule, which is the product of the degrees of membership of the observation that gave rise to it, and the rule that maximises this value in the case of conflict is selected while the rest are discarded. In this way, the initial set of rules is greatly reduced. In this particular case, the knowledge base of Expert System 1 goes from 130 to 12 rules, that of Expert System 2 from 130 to 10 rules and that of Expert System 3 from 130 to 8 rules.
- Step 4—Construction of the combined fuzzy knowledge base: after the automatic generation of the knowledge base, the need to integrate linguistic rules proposed by experts could be identified. The experts should propose the rule together with its degree of importance, so that, in conflict situations, as observed in the previous stage, the rule with the higher coefficient is given priority. In this case, no rules other than those generated in Step 3 were added.
- Step 5—Inference: Once the knowledge bases have been established, it is possible to analyze data from new patients and obtain the risk indicators at the output of each of the expert systems. These risk indicators initially vary between 0 and 1; however, to facilitate interpretation of the risk at the output of the cascade, the *Dyspnea Risk* at 12 months is scaled between 0 and 100.

##### Prof Test Results and Determination of Optimum Threshold Value

After the definition of the knowledge bases, this section deals with the analysis of the predictive and generalisation capacity of the system on the dataset reserved for testing. This dataset contains 55 patients that were not used in the knowledge base construction.

**Figure 5:**
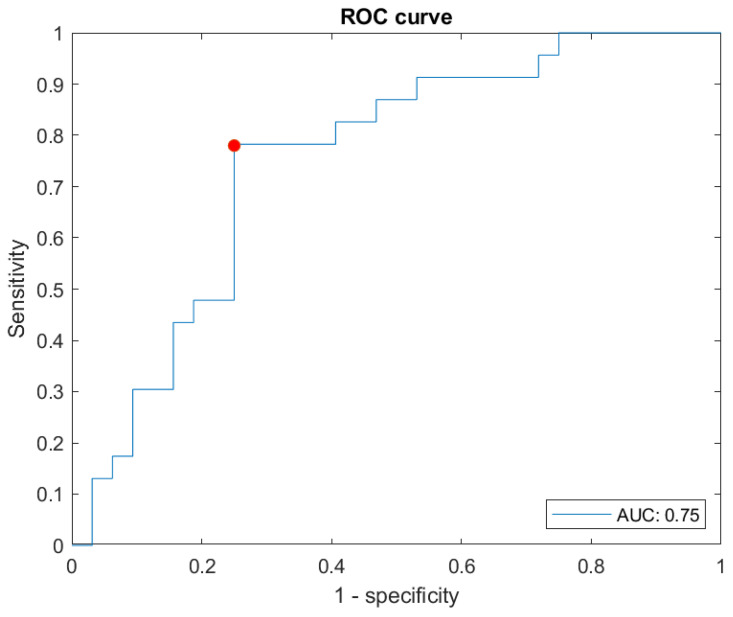
ROC curve over the test data set. The red dot indicates the point that optimises the classification values obtained after the process of optimising the Matthews correlation coefficient.

Figure 5 below shows the ROC curve obtained, supported by an AUC value of 0.75.

In order to be able to interpret the risk obtained at the cascade output, it is necessary to establish a threshold that allows discrimination between cases with ‘dyspnea at 12 months’ and cases with ‘no dyspnea at 12 months’. To achieve this, an optimisation process is performed using the test dataset to select the threshold that maximises the associated Matthews correlation coefficient (*Mcc*) [69,70,71] (Equation (1) shows its equation; the acronyms in the equation include *TN* for true negatives, *FN* for false negatives, *TP* for true positives and *FP* for false positives).

(1) $$Mcc=\frac{TN\cdot TP-FN\cdot FP}{\sqrt{\left(TP+FP)\cdot (TP+FN)\cdot (TN+FP)\cdot (TN+FN\right)}}$$

**Figure 6:**
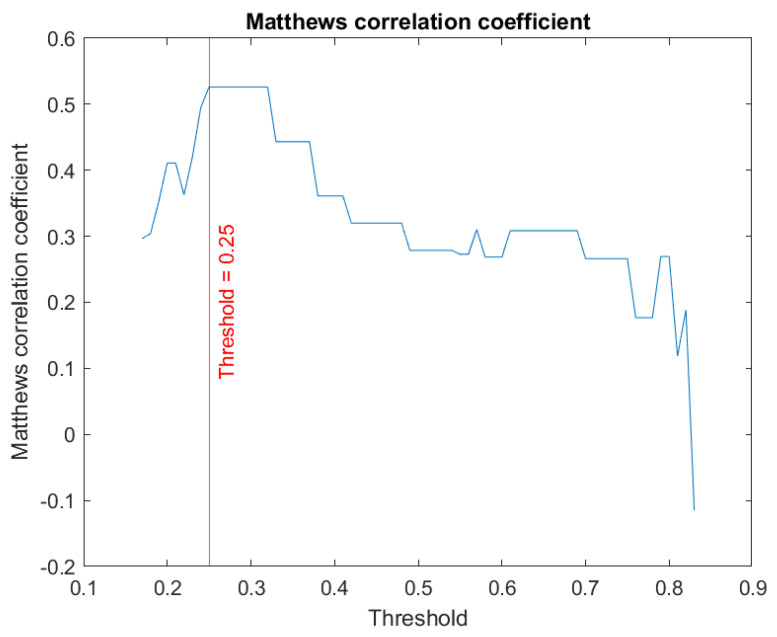
Determination of the optimal cut-off point based on Mattews correlation coefficient.

Figure 6 shows a graph of *Mcc* for the different thresholds, highlighting the threshold associated with the optimum point. At the optimum point, *Mcc* is 0.53.

Likewise, associated with this point, there is a sensitivity, also known as recall, of 0.78 and a specificity of 0.75, as can be seen in the point highlighted in Figure 5. In addition to this, an accuracy of 0.76, and a F1-score de 0.73 are presented.

#### 2.2.3. Alert Generation and Decision-Making

After processing the data, the system will suggest either ‘*dyspnea at 12 months*’ or ‘*no dyspnea at 12 months*’. This information is displayed in Panel (3), *Alert Generation and decision-making*, in Figure 2.

The medical team can use this recommendation to determine which patients require follow-up and perform personalised studies to assess their condition and evolution.

## 3. Case Study

This section presents a practical application case as a proof of concept to exemplify the operation of the intelligent system and highlight its simplicity in clinical practice. It is important to note that the case study presented was not used in the construction of the intelligent system and that this work does not aim to provide an intensive clinical validation of the system.

### 3.1. Initial Data Collection

Table 3 presents the data of a patient who was admitted for pneumonia after testing positive for COVID-19. The patient was interviewed three and twelve months after the episode and presented dyspnea on both occasions.

### 3.2. Data Processing

**Figure 7:**
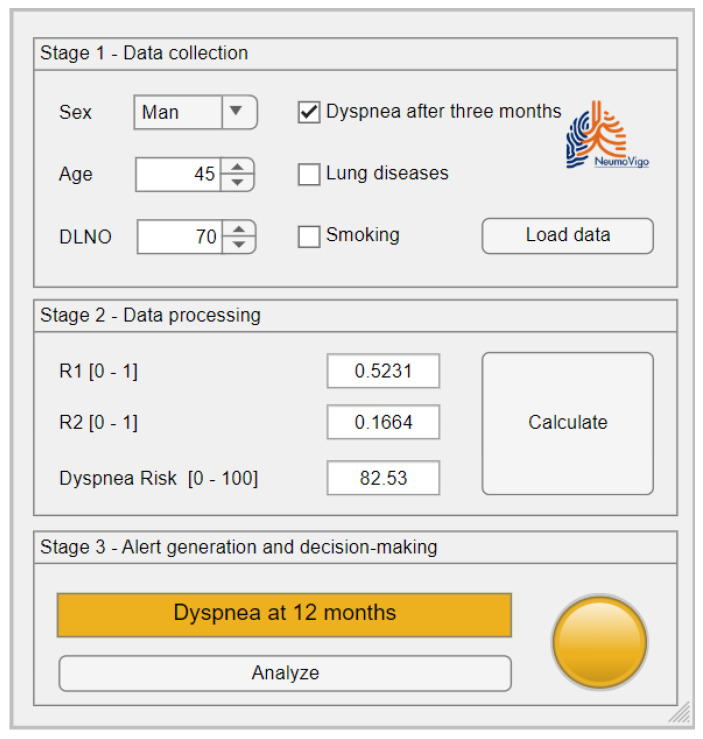
Screenshot of the results obtained in the case study.

After collecting the patient’s data, the data is entered into the application for processing by the intelligent system, as shown in Figure 7.

Table 4 presents a summary of the rules that have been fired in each of the expert systems for this case study. For more information on the membership functions and their various sections, refer to Figure 4.

Considering the input data, at the output of the first expert system we obtain R_1_, which has a value of 0.5231; concurrently, at the output of the second expert system, we obtain R_2_, which has a value of 0.1664; finally, considering R_1_ and R_2_, at the output of Expert System 3, we obtain the *Dyspnea Risk* at 12 months, which has a value of 82.53.

### 3.3. Alert Generation and Decision-Making

After determining the 12-month *Dyspnea Risk*, we proceed to interpret the results. In this case, the value exceeds the previously determined threshold, classifying the patient as a possible case of dyspnea at 12 months. This alert is visible in the third panel of Figure 7, alongside the luminous indicators.

The system-generated recommendation aligns with the current situation. During the consultation three months after the COVID-19 episode, professionals may decide to establish a personalised follow-up process with individualised specific studies.

## 4. Discussion

From the beginning of the pandemic to the present, it has been observed that a considerable number of patients have experienced persistence of COVID-19 associated symptoms for prolonged periods of time after the acute phase of the pathology, which has led to a notable increase in the demand for medical consultations and studies. This condition, known as long COVID, has been studied extensively, but determining how patients will progress or when the symptoms will return, among other possible questions, is complex. In addition, since it is a relatively recent condition, specialists often face difficulties in treating patients, as they do not have specific treatments or therapies for the condition. All of this is a major problem, especially for patients, as in many cases it can prevent them from performing their previous tasks, leaving them incapacitated.

Recently, tools supported by artificial intelligence techniques have been developed to assist specialists in identifying patients who are susceptible to developing long COVID. These tools explore long COVID in a general way, considering many symptoms together, which, for specialists in specific fields, such as pulmonology, may not be very useful. These tools are generally based on inferential approaches based on statistical learning and do not address long-term predictions (e.g., 12 months). Although long COVID encompasses a wide variety of symptoms, this paper focuses specifically on dyspnea and whether it will persist 12 months after the COVID-19 episode. Dyspnea is a prominent symptom that poses significant challenges for patients. To aid in its detection, an intelligent clinical decision support system is proposed that deploys a cascade of expert systems. We will first discuss the benefits of the proposed architecture, highlighting its ability to represent knowledge. We will then consider the clinical utility and potential relevance of the system.

The proposed system focuses on the determination of the *Dyspnea Risk* at 12 months and is based on the use of expert systems. Expert systems are one of the main representatives of the symbolic deductive reasoning of artificial intelligence and stand out for their ability to formalise and diversify knowledge. In this work, expert systems are deployed through a cascade that facilitates the compartmentalisation and management of information, resulting in the definition of simpler rules. It is important to note that the initial set of variables was reduced using a decision tree in order to determine the variables with greater predictive power. This is a practice widely applied in the current literature and facilitates the subsequent creation of simpler rules. As embedded feature selection methods, decision trees offer significant advantages over other approaches. Not only do they select the optimal set of variables during model building, but they also prove to be computationally more efficient than wrapper methods, which require multiple iterations to evaluate different subsets of variables. Unlike filter methods, they rely on model-specific statistical metrics, such as the Gini index in CART-type decision trees. However, it is crucial to recognise that, in the context of this work, other strategies for feature selection could be also considered. In this sense, the field of feature selection is a constantly evolving one, where new techniques and methods are frequently proposed, including the use of metaheuristics together with wrapper methods [72], which can offer new perspectives for feature selection, resulting in more efficient and accurate processes, or even hybrid methods that combine the advantages of filtering and wrapper approaches [73].

The definition of rules is a fundamental task (as it seeks to encapsulate the knowledge of events occurring in similar situations) and the reasoning performed by the system will depend on them. It is obvious that the rules will depend on the experts who define them; however, in this case, and given the relative novelty of this condition, there is a lack of explicit knowledge to determine these rules and define the evolution of an acute COVID-19 patient, particularly regarding the possible appearance of dyspnea 12 months later. Considering the existence of huge amounts of data on COVID-19, and given the absence of knowledge, the dilemma arises on whether to employ knowledge-based approaches or learning-based approaches. Unlike learning-based models, knowledge expressed through deductive rules represents a permanent entity that can be enriched with new knowledge. In contrast, data are volatile, a mere quantitative representation of a variable, with no greater meaning or relevance.

In this case, to solve this problem, we opted for the use of the Wang-Mendel automatic rule generation algorithm [52] so that, starting from a set of data, it is possible to create a knowledge base, expressing the different variables as symbols and relating them through rules supported by a logical structure. This is one of the main contributions and novelties of this work, and it clearly highlights the advantages of this approach, as it enables the automatic creation of knowledge bases from numerical data. In this way, the difficulty associated with the construction of knowledge bases is significantly reduced. In any case, it is also necessary to assume that these knowledge bases need to be reviewed by experts prior to their consolidation and diversification among other clinicians.

The tests performed indicate satisfactory results, supported by AUC values of 0.75 and Mcc of 0.53, which are comparable to those obtained by other proposals which are the state of the art. However, our approach has two distinctive features: the prediction is made twelve months after the COVID-19 episode, and it is also explainable.

Although our system uses models to automatically generate fuzzy rules from data, it also has the ability to explain the inference process, similar to traditional knowledge-based models. This is a fundamental and highly relevant aspect that affects the inherent explainability of the proposal and encourages the transmission and diversification of knowledge. However, again, it is important to note that the knowledge base must be reviewed by experts for further consolidation. This is not too complex in this case, given the number of rules obtained.

It is also important to assume that knowledge may have been lost during the rule generation process. This is due to the way in which the Wang-Mendel algorithm prioritises the rules (it assigns each rule obtained from the data a coefficient, resulting from the product of the degrees of membership of the antecedents and consequents of each rule). This will be an issue that will need to be addressed in the future in order to reduce the possible loss of knowledge associated with the rule generation process.

Beyond the technical aspects, it is crucial to highlight those aspects that are clinically relevant. Using this system, and with a reduced set of input variables, clinicians have a tool that allows them to assess the risk that a patient may experience dyspnea 12 months after an acute COVID-19 episode. This measure is very useful in identifying patients who are prone to long-term problems. This approach can help pulmonologists identify patients who require more intensive follow-ups and investigation and those who do not, resulting in significant savings in resources. Furthermore, the implementation of this system contributes to the advancement towards the standardisation of the diagnostic process in the context of long COVID. The introduction of a tool based on expert systems not only facilitates long-term risk assessment, but also establishes a more consistent and uniform framework for the diagnosis of this type of patients. Beyond the encouraging results obtained, it is important to note that this system is still in the early stages of development, so in the future it will be necessary to undertake intensive clinical validation to demonstrate its practical utility.

### Relevance of the Proposal

To facilitate the understanding of the benefits of the proposed system, Table 5 presents a benchmarking based on the following criteria: reasoning (understood as the system’s capacity to perform symbolic reasoning); scalability (understood as the capacity to replace/modify the system’s engines); efficiency (refers to the reliability of the results, understood through the system’s capacity to manage uncertainty) and data dependence.

In general, considering the works analyzed in Table 5, the few approaches proposed in the current literature are based on the use of statistical learning techniques, particularly machine learning. In contrast, symbolic inference presents important benefits associated with the formalisation and diversification of knowledge, as well as with the management of uncertainty. In addition, the inclusion of automatic rule generation techniques facilitates the use of symbolic approaches. Considering all these issues, the proposed system represents a clear novelty in the diagnostic field of dyspnea after an episode of severe COVID-19, facilitating this arduous task for specialists in this area.

## 5. Conclusions

In this paper we presented the proposal of an intelligent system applied to the prediction of dyspnea 12 months after cases of severe COVID-19. Unlike other common works in the current literature, a unique approach is addressed based on the use of a cascade of expert systems whose knowledge bases were generated through the Wang-Mendel automatic rule generation approach.

The operation of the system was exemplified in the case study section as a proof of concept with the aim of demonstrating its potential in clinical practice and its ease of use. In any case, and despite the results obtained (supported by AUC values close to 0.8), it is important to note that this system is in early stages of development.

Regarding the limitations of this work, it is important to recognise that the size of the database used is relatively small, which could limit the generalisability of the results obtained. Furthermore, from an architectural point of view, and with regard to the Wang-Mendel automatic rule generation algorithm more specifically, the need to review and improve the rule selection method is identified in order to avoid possible loss of knowledge. It is also important to point out the need for expert reviews of the knowledge bases before final consolidation. This process, although tedious, is very beneficial, and its main objective is to enable the consolidation and subsequent diversification of the knowledge base, thus ensuring the reliability and trustworthiness of the recommendations provided by the system.

In the future, it will be essential to carry out an intensive clinical validation of the system to adapt its use to routine practice and to test its usefulness in real-life settings. Expanding the database to include new cases will be a priority in improving the robustness and generalisability of the system.

## Figures and Tables

**Table 1:** Summary of the variables.

	Group	Variable	Type	Comment
**Independent variables**	General and anthropometric data	Sex	Categorical	Man (1)/woman (0)
		Age	Numerical	-
		Body mass index (BMI)	Numerical	-
	Toxic habits	Smoking	Categorical	Yes(1)/no(0)
	Previous comorbidities	Lung diseases	Categorical	Yes(1)/no(0)
		Heart diseases	Categorical	Yes(1)/no(0)
		Diabetes	Categorical	Yes(1)/no(0)
		Arterial hypertension	Categorical	Yes(1)/no(0)
	Severity of the disease	Severity of pneumonia	Categorical	Refers to the unit to which the patient was admitted during hospitalisation for COVID-19-associated pneumonia: ICU(1)/IRCU(0).
	Situation after three months	Dyspnea after three months	Categorical	The Modified British Medical Research Council (mMRC) scale is used to assess dyspnea. If the score is zero, the patient has no dyspnea; if the score is greater than zero, the patient has symptoms of dyspnea. Yes(1)/No(0)
		Chest X-ray	Categorical	Affected X-ray (1)/No affect X-ray (0)
		Alteration of pulmonary diffusion: DLNO	Numerical	Reference percentage of the diffusion capacity in relation to the theoretical value
**Dependent variable**	Situation after 12 months	Dyspnea after twelve months	Categorical	The Modified British Medical Research Council (mMRC) scale is used to assess dyspnea. If the score is zero, the patient has no dyspnea; if the score is greater than zero, the patient has symptoms of dyspnea. Yes (1)/No (0)

**Table 2:** Summary of input and output variables for each expert system.

**Expert System 1**	
Input	Dyspnea at 3 months, Sex and Age
Output	R_1_
**Expert System 2**	
Input	Lung diseases, Smoking and DLNO
Output	R_2_
**Expert System 3**	
Input	R_1_ and R_2_
Output	*Dyspnea Risk* at 12 months

**Table 3:** Data of the patient to study.

Variable	Value
Sex	Man
Age	45
Dyspnea at third month	Yes
Lung diseases	No
Smoking	No
DLNO	70

**Table 4:** Summary of the rules fired in the case study.

Expert System	Rules
Expert System 1	IF (Dyspnea at third month is Yes) AND (Age is Low) AND (Sex is Man) THEN (R_1_ is Dyspnea) IF (Dyspnea at third month is Yes) AND (Age is Medium) AND (Sex is Man) THEN (R_1_ is Dyspnea)
Expert System 2	IF (Lung diseases is No) AND (Smoking is No) AND (DLNO es medium) THEN (R_2_ is No Dyspnea) IF (Lung diseases is No) AND (Smoking is No) AND (DLNO es High) THEN (R_2_ is No Dyspnea)
Expert System 3	IF (R_1_ is Medium) AND (R_2_ is Low) THEN (R_3_ is Dyspnea) IF (R_1_ is High) AND (R_2_ is Low) THEN (R_3_ is Dyspnea)

**Table 5:** Benchmarking. Our proposal is compared with other state-of-the-art works according to four essential criteria (reasoning, scalability, efficiency and data dependence). For each state-of-the-art work, a symbol is associated with each of the criteria. The symbol “-” indicates that the state-of-the-art proposal has inferior characteristics compared to our proposal considering the criterion. The symbol “=” indicates that the state-of-the-art proposal has similar characteristics to our proposal considering the criterion.

	Reasoning	Scalability	Efficiency	Data Dependence
Plaff et al. [47]	The system relies on statistical inference approaches.	The system is not scalable.	The authors employed XGBoost model for their analysis. They used an implicit approach to manage uncertainty based on probabilities.	This is a fully data-dependent approach, as it employs supervised learning approaches.
	-	-	=	=
Blessy Antony et al. [48]	The system relies on statistical inference approaches.	The system is not scalable.	The authors employed logistic regression and random forest for their analysis. They used an implicit approach to manage uncertainty based on probabilities.	This is a fully data-dependent approach, as it employs supervised learning approaches.
	-	-	=	=
Reme et al. [49]	The system relies on statistical inference approaches.	The system is not scalable.	The authors employed the LASSO and random forest models to analyse the data. They also utilised an implicit approach to manage uncertainty based on probabilities.	This is a fully data-dependent approach, as it employs supervised learning approaches.
	-	-	=	=
Gupta et al. [50]	The system relies on statistical inference approaches.	The system is not scalable.	The authors used a stacking ensemble model supported by Deep Neural Networks to analyse the data. They also utilised an implicit approach to manage uncertainty based on probabilities.	This is a fully data-dependent approach, as it employs supervised learning approaches.
	-	-	=	=
Patel et al. [51]	The system relies on statistical inference approaches.	The system is not scalable.	The authors employ a Random Forest model to analyse the data. They also utilised an implicit approach to manage uncertainty based on probabilities.	This is a fully data-dependent approach, as it employs supervised learning approaches.
	-	-	=	=
Our proposal	The proposed system relies on the use of symbolic inference approaches.	The proposed system is scalable, since it is possible to modify the inference engines.	The system uses fuzzy inference engines, which allow uncertainty management from a non-probabilistic point of view.	Data dependence is present, since they are necessary to define the knowledge bases of the expert systems.

## Data Availability

The data presented in this study are available on request from the corresponding author.
